# In a three-dimensional reconstructed human epidermis filaggrin-2 is essential for proper cornification

**DOI:** 10.1038/cddis.2015.29

**Published:** 2015-02-19

**Authors:** V Pendaries, M Le Lamer, L Cau, B Hansmann, J Malaisse, S Kezic, G Serre, M Simon

**Affiliations:** 1 UMR5165 CNRS, CHU Purpan, Place du Dr Baylac TSA40031, 31059 Toulouse CEDEX 9, France; 2 U1056 INSERM, CHU Purpan, Place du Dr Baylac TSA40031, 31059 Toulouse CEDEX 9, France; 3 University of Toulouse, CHU Purpan, Place du Dr Baylac TSA40031, 31059 Toulouse CEDEX 9, France; 4 Department of Dermatology, University Hospital of Schleswig-Holstein, Schittenhelmstrasse 7, 24105 Kiel, Germany; 5 Cell and Tissue Laboratory, URPHYM-NARILIS, University of Namur, Rue de Bruxelles, 61, 5000 Namur, Belgium; 6 Academic Medical Center, Coronel Institute of Occupational Health, PO Box 22 700, 1100 DE Amsterdam, The Netherlands

## Abstract

Atopic dermatitis is a chronic inflammatory skin disease with defects in the epidermal barrier. In a cohort of African-American children, a *FLG2* nonsense mutation has been associated with the disease. In the epidermis of European patients, the expression of filaggrin-2, the filaggrin-related protein encoded by *FLG2*, is decreased. To describe the function of filaggrin-2 and evaluate the impact of its deficiency, its expression was downregulated using lentivirus-mediated shRNA interference in a three-dimensional reconstructed human epidermis (RHE) model. This resulted in parakeratosis and a compact *stratum corneum*, presence of abnormal vesicles inside the corneocytes, increased pH and reduced amounts of free amino acids at the RHE surface, leading to increased sensitivity to UVB radiations. The expression of differentiation markers was slightly modified. However, we observed reduced proteolytic processing of corneodesmosin, hornerin and filaggrin in parallel with reduced amounts of caspase-14 and bleomycin hydrolase. Our data demonstrated that filaggrin-2 is important for a proper cornification and a functional *stratum corneum*. Its downregulation in atopic patients may be involved in the disease-associated epidermis impairment.

Atopic dermatitis (AD; OMIM #603165), also known as atopic eczema, is a very common inflammatory skin disease.^1, 2^ It is the result of complex interactions between genetic and environmental factors. The most robust and widely replicated genetic risk factor for the disease corresponds to nonsense mutations of the gene *FLG.*^3, 4^ This gene encodes filaggrin, an S100 fused-type protein essential for the epidermal barrier functions.^3, 4, 5^ Filaggrin deficiency is responsible for decreased amounts of free amino acids in the *stratum corneum* (SC),^6, 7, 8, 9^ abnormal keratinocyte differentiation,^6, 10^ epidermal barrier defects and enhanced percutaneous sensitization,^6, 7, 9^ all characteristics of the atopic skin. However, a significant number of Asian and European patients with AD do not display any of the numerous known *FLG* mutations,^3, 4^ in particular in the South of Europe.^11, 12^ In addition, *FLG* nonsense mutations have not been detected in Ethiopian and South African populations,^13, 14^ and are not associated with AD in African-American patients.^15, 16^ Hence, the defect in epidermal barrier functions of patients without *FLG* mutations, including those of African ancestry, may be associated with other inherited/acquired abnormalities that compromise keratinocyte differentiation.^17^

Recently, mutations in the *FLG2* gene, in particular a nonsense mutation, were shown to be associated with persistent AD in a cohort of 60 US patients of African ancestry.^18^ *FLG2* encodes filaggrin-2, another S100 fused-type protein.^19^ Filaggrin-2 is very similar to filaggrin in terms of protein structure, amino-acid composition, pattern of expression and of biochemical properties. It is synthetized by granular keratinocytes as a large precursor consisting of 23 homologous repeats and a S100-homologous N-terminal domain. Filaggrin-2 and filaggrin are colocalized in keratohyalin granules in granular keratinocytes, and in the cytoplasmic matrix of the lower corneocytes.^20, 21^ In the upper SC, filaggrin-2 is deiminated and degraded by calpain 1.^20^ The role of filaggrin-2 in the SC remains to be discovered, but it may be similar to that of filaggrin.^22^ In addition, *FLG2* is one of the genes that are the most downregulated after cholesterol depletion of keratinocytes, an experimental model of AD.^23^ Importantly, we and others have shown that filaggrin-2 expression is reduced, probably by pro-inflammatory cytokines, in the epidermis of European patients.^12, 24^

To analyze the function of filaggrin-2 and understand the effect of its deficiency in AD in an immunological cell-free context, we downregulated its expression with shRNA technology in reconstructed human epidermis (RHE). This technology has previously been used with success to demonstrate the importance of filaggrin in the human epidermis.^6^ The research focused particularly on epidermal differentiation, SC properties and permeability barrier.

## Results

### Filaggrin-2 knockdown in RHE leads to a thinner epidermis, parakeratosis, a compact SC and the presence of abnormal vesicles inside corneocytes

Filaggrin-2 expression knockdown in RHE was achieved using RNA interference in normal human primary keratinocytes that were secondarily seeded on polycarbonate membranes and differentiated at the air–liquid interface for 10 days, as previously described.^6, 25^ This was performed with keratinocytes obtained from three different donors. Two shRNAs targeting filaggrin-2 (shFLG2a and shFLG2b) were used separately.

**Figure 1:**
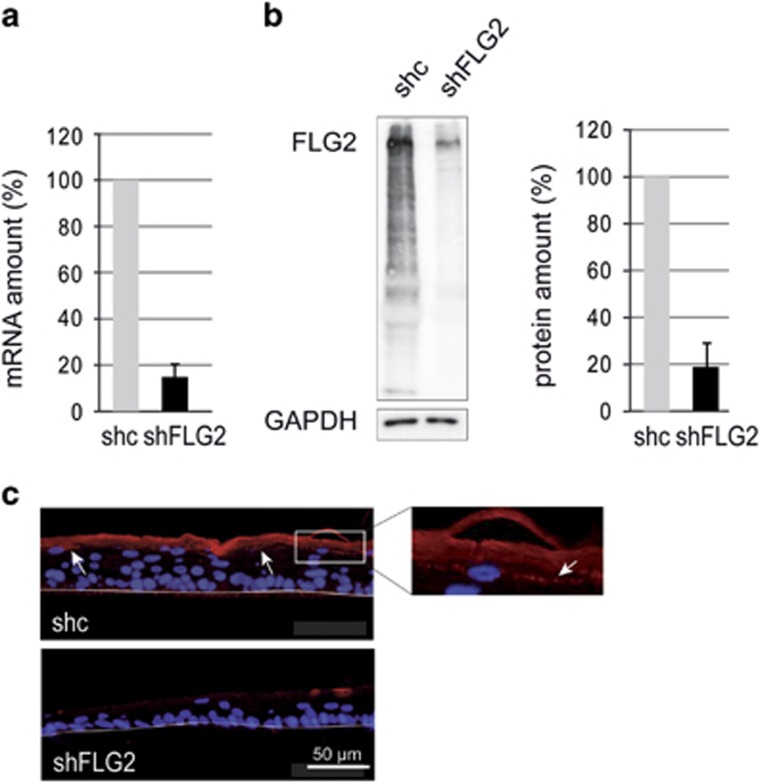
Expression of filaggrin-2 is efficiently inhibited. (**a**) At day 10, fully differentiated shc- and shFLG2-RHEs were analyzed by qRT-PCR. The expression of the TATA box-binding protein gene was used for normalization. (**b**) Identical shc- and shFLG2-RHEs were analyzed by western blot with a polyclonal antibody specific for the spacer domain of filaggrin-2 (FLG2). A representative result is shown on the left. Immunodetected filaggrin-2 was quantified and normalized to glyceraldehyde 3-phosphate dehydrogenase (GAPDH) levels (shown on the right). (**c**) Identical shc- and shFLG2-RHEs were analyzed by immunofluorescence with the same antibody. In shc-RHE, filaggrin-2 was detected in the *stratum granulosum* with a granular labeling (arrows and insert) and in the *stratum corneum*. The polycarbonate filter–epidermal junction is indicated by a thin line. The mRNA and protein amounts corresponding to shc-RHE were arbitrarily set at 100. The error bars correspond to the S.D. calculated from independent experiments performed with keratinocytes from three different donors, one being duplicated for qRT-PCR (*n*=4) and each being duplicated for western blotting (*n*=6)

After 10 days of culture at the air–liquid interface of either normal keratinocytes or keratinocytes transduced with a control shRNA that does not target any known human gene, morphologically differentiated RHEs were obtained. Proper expression of differentiation markers and a functionally competent SC were achieved, as reported before.^6^ In particular, filaggrin and filaggrin-2 were detected in the *stratum granulosum* with a granular pattern and in the SC (Supplementary Figure S1a and Figure 1c). Similar locations are known in the native epidermis, where the two proteins are located in the keratohyalin granules and the corneocyte matrix.^19, 20, 21^ The RHEs produced with keratinocytes transduced with either the control shRNA (shc-RHE) or shFLG2a (shFLG2-RHE) were analyzed by reverse transcription-quantitative PCR (RT-qPCR) and western blotting with a polyclonal antibody directed to filaggrin-2 (Figures 1a and b). In shFLG2-RHE, filaggrin-2 mRNA and protein amounts were decreased by 85% and 78%, respectively, compared with shc-RHE. Although similar but not identical levels of filaggrin-2 expression were observed in the RHEs produced with keratinocytes from the three different donors (Supplementary Figures S1b and c), the efficacy of downregulation was the same. Immunofluorescence labeling of RHE sections showed the expression of filaggrin-2 in the granular layers and SC. Confirming the efficacy of downregulation, the staining of shFLG2-RHE was markedly reduced (Figure 1c). Similar results were obtained with shFLG2b (illustrated in Supplementary Figure S2).

**Figure 2:**
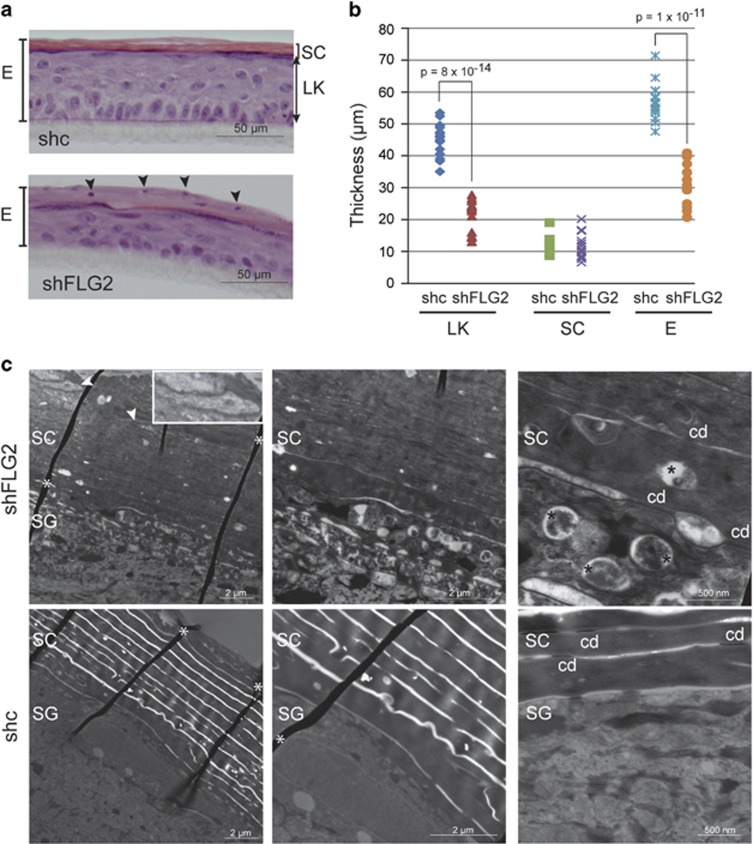
Filaggrin-2 deficiency induces alteration in the epidermal morphology. (**a**) Stained sections of shc- and shFLG2-RHEs are shown (one representative experiment of three). (**b**) Thickness of the living nucleated keratinocyte layers (LK), the *stratum corneum* (SC) and the total epidermis (E) were measured on images of five independent couples of RHEs produced with keratinocytes from three different subjects. Differences in thickness values (*n*=15) were analyzed using Student's *t*-test. (**c**) RHEs were analyzed by electron microscopy. Corneodesmosomes (cd), nuclear remnants (arrowheads and insert) and abnormal vesicles in the corneocyte matrix (black stars) are shown. Folded areas of the sections are indicated by white asterisks. SG, *stratum granulosum*

At a morphological level, shFLG2-RHE was thinner than shc-RHE with a reduced thickness of the living keratinocyte layers, as shown by hematoxylin–eosin staining (Figures 2a and b). The SC thickness was identical, but parakeratosis was evident in the shFLG2-RHE. To try to understand this effect on epidermis thickness, the keratinocyte proliferation rate was analyzed. Before epidermal reconstruction, no modification was observed. However, in RHE at day 10, a significant increase in Ki67 staining was observed (Supplementary Figure S3). This indicated that the reduced thickness was not due to reduced proliferation of the shFLG2-treated keratinocytes.

Transmission electron microscopy analysis of shc- and shFLG2-RHE (Figure 2c) showed similar keratinocyte organization and structures, including desmosomes, keratohyalin granules and corneodesmosomes. However, at the lower magnification, parakeratosis of shFLG2-RHE was confirmed and the SC appeared denser without clear extracellular spaces. At the highest magnification, abnormal material was visible in the extracellular spaces, and the corneocytes displayed unusual, large, intracellular vesicles.

### The amino-acid content and pH of the upper SC are disturbed in filaggrin-2-deficient RHE

**Figure 3:**
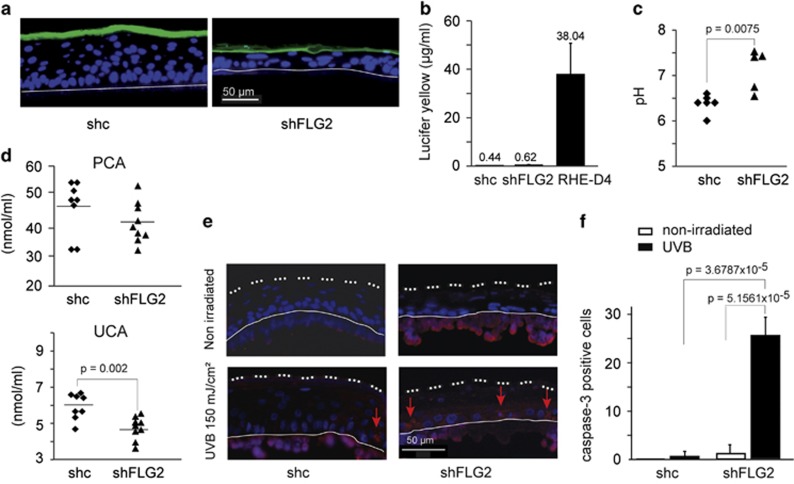
Filaggrin-2 knockdown leads to impaired *stratum corneum* properties. (**a** and **b**) A solution of Lucifer yellow was applied to the shc- and shFLG2-RHEs. After 6 h of incubation, location of the dye was investigated using a fluorescence microscope (**a**) and dye concentration in the culture medium was quantified (**b**). As a control, a normal RHE at day 4 was used (RHE-D4). (**c**) The pH was measured at the surface of the RHEs. Each indicated value corresponds to the mean pH of a different RHE (*n*=5 shc-RHEs and *n*=6 shFLG2-RHEs). The RHEs were produced with keratinocytes from two different donors. (**d**) Pyrrolidone carboxylic acid (PCA) and urocanic acid (UCA) amounts were quantified in lysates of shc-RHEs (*n*=8) and shFLG2-RHEs (*n*=9). RHEs were produced with keratinocytes from the same two different donors. (**e**) Active caspase-3 (red staining; arrows) was detected without and with UVB irradiation, as indicated. Please note the nonspecific labeling of the polycarbonate membrane (under the thin line). (**f**) The number of active caspase-3-positive cells was quantified and is indicated per RHE length unit. Differences in pH, in amino-acid amounts and in number of cells were analyzed using Student's *t*-test. Only statistically significant differences are indicated

To test for a possible effect of filaggrin-2 knockdown on SC permeability, we used the Lucifer yellow assay.^9^ At day 10, the hydrophilic fluorescent dye was applied to the external surface of shc- and shFLG2-RHE. As observed by fluorescence microscopy (Figure 3a), the green dye was retained in the SC of both control and filaggrin-2-deficient RHEs. In addition, Lucifer yellow was hardly detectable in the culture medium (Figure 3b). In contrast, large amounts of the dye were detected when RHEs were analyzed at day 4 as a control. This indicated that the outside-in permeability was not affected by the deficiency in filaggrin-2.

Similar to filaggrin, filaggrin-2 has been suspected to be degraded to free amino acids in the upper SC. These amino acids and their derivatives may be involved in the acidification of the SC and in the formation of natural moisturizing factor. Therefore, we measured the pH at the external surface of the shc- and shFLG2-RHE (Figure 3c). Filaggrin-2 inhibition induced a statistically significant pH increase (mean value from 6.38±0.25 to 7.1±0.15; *P*=0.0075). Then, we quantified the contents of the urocanic acid and pyrrolidone carboxylic acid in the RHE. HPLC analysis of RHE lysates (Figure 3d) revealed a statistically significant urocanic acid decrease in filaggrin-2-knockdown RHE as compared with the control samples (from 5.90 to 4.80 mmol/ml). Concerning the pyrrolidone carboxylic acid concentration, a similar trend was observed (from 47.66 to 40.68 mmol/ml) but the decrease did not reach statistical significance. As urocanic acid is known to protect skin against UVB radiation, we irradiated shc- and shFLG2-RHE and detected active caspase-3 as a marker of apoptosis, as previously described.^6^ Downregulation of filaggrin-2 resulted in an increase in caspase-3 activation, showing increased radiation sensitivity (Figures 3e and f).

### Filaggrin-2 deficiency alters the epidermal differentiation program

**Figure 4:**
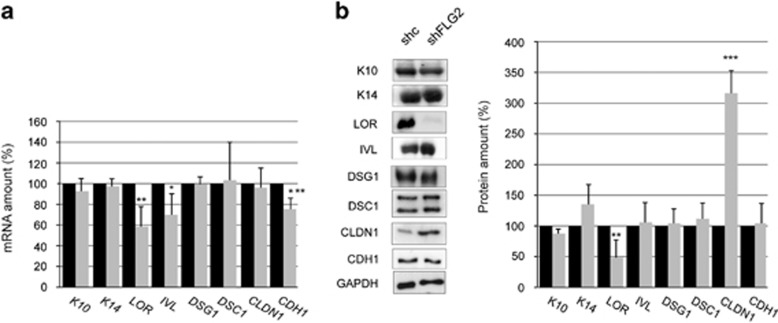
Filaggrin-2 deficiency affects keratinocyte differentiation. (**a**) Expression of keratin (K)14 and differentiation-associated genes was analyzed by qRT-PCR in shc-RHEs (black bars) and shFLG2-RHEs (gray bars). The expression of the TATA box-binding protein gene was used for normalization. *P=0.041; **P=0.005; ***P=0.0036. (**b**) Expression of the corresponding proteins was analyzed by western blotting, quantified and normalized to glyceraldehyde 3-phosphate dehydrogenase (GAPDH) immunoreactivity. **P=0.0039; ***P=5 × 10^−4^. The mRNA and protein amounts corresponding to shc-RHE were arbitrarily set at 100. Mean values and S.D. (error bars) were calculated from independent experiments performed with keratinocytes from three different donors, each being duplicated (*n*=6). Data were compared using Student's *t*-test. Only statistically significant differences are indicated. LOR, loricrin; IVL, involucrin; DSG1, desmoglein 1; DSC1, desmocollin 1; CLDN1, claudin 1; CDH1, E-cadherin

In order to test for a possible effect on keratinocyte differentiation, the expression of a panel of genes differentially expressed in the various layers of the epidermis was explored at the mRNA level by quantitative real-time PCR (Figure 4a). As compared with shc-RHE samples, mRNA levels of loricrin, involucrin and E-cadherin were slightly reduced in filaggrin-2-deficient RHE. The levels of mRNAs encoding keratin (K)14, K10, desmoglein 1, desmocollin 1 and claudin 1 were not modified. To determine whether the modifications at the mRNA level translated to the protein level, western blotting was performed and the detected bands were quantified (Figure 4b). Compared with controls, the detection of loricrin was decreased in shFLG2-RHE. Interestingly, the immunodetected claudin 1 amount was increased by a factor of 3. The expression of K14, K10, involucrin, desmoglein 1/2, desmocollin 1 and E-cadherin was not modified.

**Figure 5:**
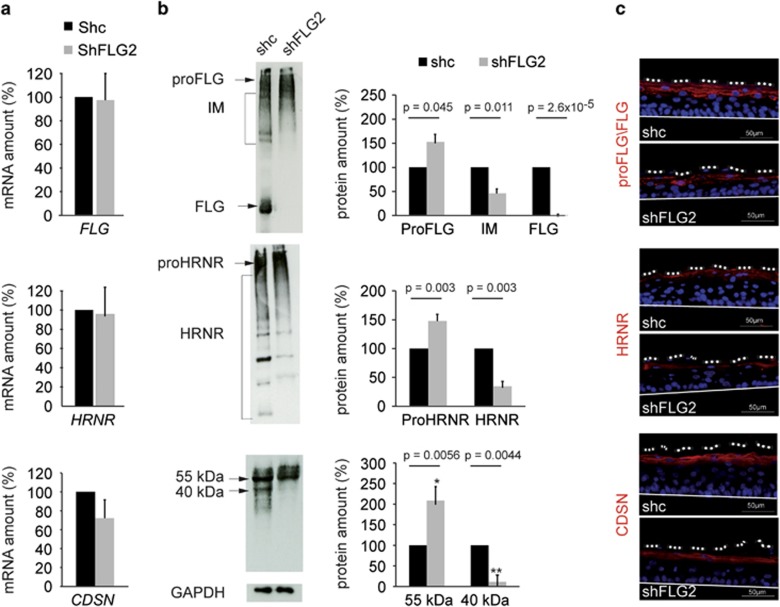
Filaggrin-2 deficiency leads to proteolytic processing defects. (**a**) Expression of filaggrin (FLG), hornerin (HRNR) and corneodesmosin (CDSN) genes was investigated by qRT-PCR in shc- and shFLG2-RHEs. (**b**) Expression of the corresponding proteins was analyzed by western blotting and the indicated bands were quantified. Error bars represent the S.D. (**c**) ProFLG/FLG, HRNR and CDSN expression patterns were investigated by immunohistology. The mRNA and protein amounts corresponding to shc-RHE were arbitrarily set at 100. Mean values and S.D. (error bars) were calculated from independent experiments performed with keratinocytes from three different donors, one being duplicated for qRT-PCR (*n*=4) and each being duplicated for western blotting (*n*=6). Data were analyzed using Student's *t*-test. Only statistically significant differences are indicated

It has been suggested that the expressions of filaggrin-2, filaggrin and hornerin are coregulated.^12^ Therefore, we investigated the expression of the last two S100 fused-type proteins (Figure 5). No changes were detected at the mRNA level, confirming the specificity of the shRNAs used (Figure 5a). When shc- and shFLG2-RHE extracts were analyzed by western blotting, different patterns of immunodetection were observed (Figure 5b). Profilaggrin was shown to accumulate whereas filaggrin monomers were not detected and intermediates decreased by 52%. Similar results were obtained concerning hornerin with an increase in the detection of the entire protein (prohornerin) and a decrease of 60% in the smaller bands. To test whether the proteolytic processing of other epidermal proteins was affected by the knockdown of filaggrin-2, we analyzed corneodesmosin in the same way (Figures 5a and b, lower part). Whereas the corneodesmosin mRNA amounts were similar, the entire 55 kDa protein increased and the 40 kDa processed form decreased by 90%. When indirect immunofluorescence analysis was performed (Figure 5c), the labeling patterns obtained with the anti-filaggrin and anti-hornerin antibodies were modified. Filaggrin and hornerin were not detected in the SC of shFLG2-RHE but only in the *stratum granulosum*, with a granular pattern suggesting an accumulation of their respective proforms in the keratohyalin granules. The distribution of corneodesmosin was not altered.

### Procaspase-14 and bleomycin hydrolase expression is reduced in shFLG2-RHE

**Figure 6:**
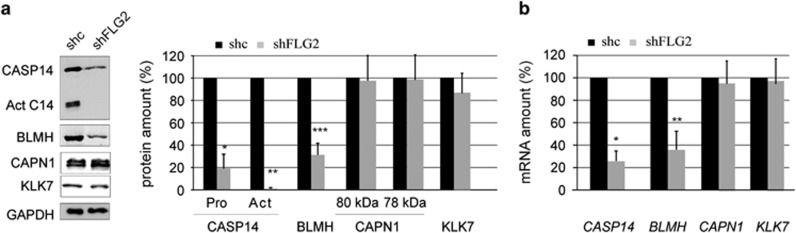
Expression of proteases is decreased in the absence of filaggrin-2. (**a**) Expression of caspase-14 (CASP14), bleomycin hydrolase (BLMH), calpain 1 (CAPN1) and kallikrein 7 (KLK7) in shc- and shFLG2-RHE was analyzed by western blotting and quantified. Act C14, active processed form of CASP14. **P=0.0004; **P=7.8 × 10^−10^; ***P=8.09 × 10^−5^. (**b**) Expression of the corresponding genes was investigated by qRT-PCR. *P=3.06 × 10^−6^; **P=1.09 × 10^−5^. The mRNA and protein amounts corresponding to shc-RHE were arbitrarily set at 100. The error bars correspond to the S.D. calculated from independent experiments performed with keratinocytes from three different donors, one being duplicated for qRT-PCR (*n*=4) and each being duplicated for western blotting (*n*=6). Data were analyzed using Student's *t*-test. Only statistically significant differences are indicated

Several proteases are known to be involved in the processing of filaggrin, hornerin and corneodesmosin, including caspase-14, bleomycin hydrolase, calpain 1 and kallikrein 7. To study the protease deficiency in shFLG2-RHE, the expression of these enzymes was analyzed by western blotting (Figure 6a). No differences were observed for calpain 1 or kallikrein 7. Procaspase-14 and bleomycin hydrolase protein levels were reduced by 81% and 69%, respectively, in shFLG2-RHE compared with shc-RHE (Figure 6a). In the shFLG2-RHE, in addition to the downregulation of procaspase-14, a reduction was observed in the processing of the proform to the active form of the protease (mean factor of 1.3±0.6; *n*=4). To determine whether the observed modifications of enzyme expression were because of changes in the corresponding mRNA levels, a quantitative real-time PCR analysis was performed (Figure 6b). Compared with control RHE samples, mRNA levels of caspase-14 and bleomycin hydrolase were significantly reduced in filaggrin-2-deficient RHE, whereas mRNA levels of calpain and kallikrein 7 were not modified.

## Discussion

Since the first description of the expression of *FLG2* in differentiated keratinocytes, little has been done to understand the role of the encoded protein, filaggrin-2. However, the close relationships between filaggrin and filaggrin-2 suggested that the two proteins could play similar roles in the epidermal barrier function.^20, 21^ The recent descriptions of an association between *FLG2* nonsense mutations and disease persistence in AD patients of African ancestry, and of a reduced expression of the protein in the epidermis of Caucasian patients, prompted us to look for filaggrin-2 function using RHE as an experimental model. We report here that filaggrin-2 knockdown by RNA interference in RHE led to alterations in the keratinocyte differentiation program. This culminated in changes of SC morphology and functions. More particularly, we observed changes in the amounts of loricrin and claudin 1, defects in the proteolytic processing of filaggrin, hornerin and corneodesmosin, and a compact SC with parakeratosis, abnormal intracellular vesicles, a higher external surface pH and decreased urocanic acid content. These data demonstrate the importance of filaggrin-2 in cornification and suggest its implication in AD pathophysiology.

Filaggrin-2 is composed of an NH2-terminal domain homologous to S100 proteins and by two central domains formed by repeated subunits. Because (1) the amino-acid composition of filaggrin-2 B-type repeats is similar to that of filaggrin, (2) filaggrin and filaggrin-2 B-type repeats are concomitantly degraded in the upper SC and (3) both proteins are substrates of calpain 1, it has been suggested that the breakdown of filaggrin-2 into free amino acids contributes to the production of natural moisturizing factor components, control of the pH at the SC surface and photoprotection of the epidermis.^20, 21^ Supporting these hypotheses, the SC pH was increased in filaggrin-2 knockdown RHE as compared with control RHE, and the urocanic acid and pyrrolidone 5-carboxylic acid contents were decreased. The decrease was not so pronounced as by the *FLG* loss-of-function mutations (i.e., one mutation results in ∼50% reduction) but is consistent with their relative contribution to the risk for AD. In addition, we observed an increased UVB-induced apoptosis of keratinocytes, urocanic acid being a major chromophore in the skin protecting from UVB radiation.^9^

Many features indicated that the keratinocyte differentiation program is perturbed when filaggrin-2 expression is downregulated. Such perturbations include a reduced expression of loricrin at both the mRNA and protein levels and an apparent stabilization of claudin 1. Many of them suggested premature cornification: a reduced thickness of spinous/granular layers without reduction of the keratinocyte proliferation rate, persistence of nuclei (parakeratosis) and abnormal vesicles in the corneocyte matrix and reduced proteolytic processing of the late differentiation proteins (hornerin, profilaggrin and corneodesmosin). An increased epidermal turnover has been suggested to induce incomplete removal of organelles because the cellular proteolytic machinery necessary for protein/nucleic acid degradation is overfed.^26^ In an alternative explanation, the expression of procaspase-14, the precursor form, and of the processed form of caspase-14, the active form of the protease, was highly reduced in shFLG2-RHE. Caspase-14 deficiency has been reported to promote parakeratosis in skin equivalent models treated with siRNA and in knockout mice.^27, 28^ Interestingly, parakeratosis has also been described in some AD patients,^29, 30^ in particular in skin regions where caspase-14 was not detected.^28^

In order to understand the above-mentioned reduced proteolytic processing of profilaggrin and corneodesmosin, we investigated the expression of the respectively suspected proteases, calpain 1 and kallikrein 7. The expression of both proteases was similar in filaggrin-2 knockdown and control RHE. Therefore, alterations in their activity and/or localization could be supposed. As the pH was increased in the filaggrin-2-knockdown epidermis, and as kallikrein 7 has been reported to be more active at neutral than acidic pH, difference in the pH is probably not the explanation. Another possibility concerning kallikrein 7 is altered secretion. Loss of filaggrin-2 led to a compact SC with apparently abnormal extracellular spaces and the presence of intracellular large vesicles, suggestive of abnormal secretion of lamellar body content. It has been discussed how mutations in *FLG*, *FATP4* and *TMEM79* genes and increase in SC pH converge to produce defective lamellar body and/or to impair the secretion of these structures in patients with AD.^17^ Mutations in *FLG2* and reduced expression of filaggrin-2 may well be involved.

**Figure 7:**
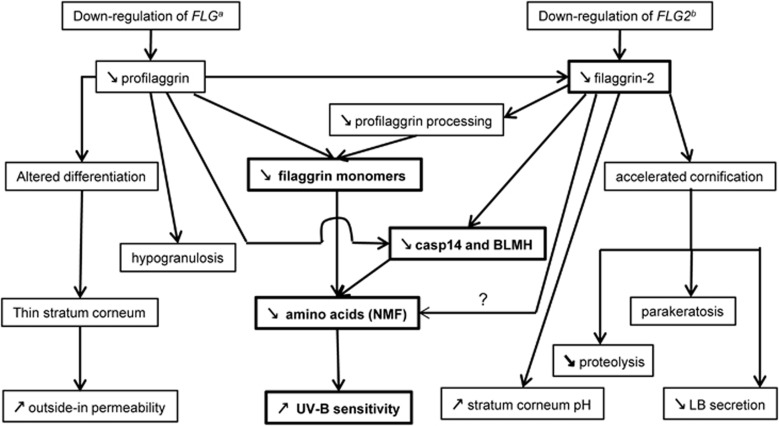
Comparison of the effects of filaggrin-2 and filaggrin knockdown using shRNAs in reconstructed human epidermis. Similar consequences are indicated in bold. BLMH, bleomycin hydrolase; FLG, filaggrin; LB, lamellar body; NMF, natural moisturizing factor. ^a^Pendaries *et al*^6^; ^b^this work

When we compared the effects of filaggrin deficiency and those of filaggrin-2 deficiency in RHE, some similarities were observed (Figure 7 and Table 1), confirming that the proteins are related. This induces a certain complexity in the interpretation of the results. For example, are the reductions in the level of natural moisturizing factor components and in the expression of loricrin, bleomycin hydrolase and caspase-14 directly because of the downregulation of filaggrin-2 or are they a consequence of the reduced production of filaggrin monomer or both? The increase of claudin-1, a tight junction protein, suggests a compensatory mechanism to overcome SC defects. However, the functions of filaggrin and filaggrin-2 are probably not identical as differences were also noted. In particular, whereas filaggrin deficiency in RHEs and in a three-dimensional skin model leads to enhanced penetration of foreign molecules,^6, 31^ filaggrin-2 knockdown appeared not to disturb outside-in SC permeability: Lucifer yellow was retained in the SC of shFLG2- as well as shc-RHE. Modification of the SC superficial pH was only observed when FLG2 was downregulated. Hypogranulosis was only evidenced when filaggrin was downregulated, even if filaggrin-2 is also a component of keratohyalin granules,^20, 21^ suggesting that the former is much more abundant than the second. Accordingly, the copy number of filaggrin mRNA per 10 ng of total RNA is ∼10^5^,^32^ and that of filaggrin-2 is between 6 and 30.^21^

Some of the effects induced by filaggrin-2-deficiency reproduced some AD-related epidermis alterations (Table 1), as already mentioned, including parakeratosis, an apparently reduced degradation of corneodesmosome components including corneodesmosin,^33^ reduced amounts of bleomycin hydrolase^34^ and natural moisturizing factor, altered profilaggrin processing^34^ and the presence of vesicles inside corneocytes.^35^ Similarly, the AD-associated increase in the SC superficial pH^36, 37^ could be because of the decrease in filaggrin-2 amount, as it has not been observed in filaggrin-null mice,^7^ filaggrin knockdown RHEs^6^ or patients with *FLG* nonsense mutations.^38^

In conclusion, this study demonstrates that filaggrin-2 is essential for normal keratinocyte differentiation, and that filaggrin-2 downregulation may be responsible for reported risk to AD.

## Materials and Methods

### shRNA lentiviral particles

For *FLG2* knockdown in keratinocytes, we used MISSION pLKO.1-puro vector based lentiviral particles containing a puromycin resistance gene and a shRNA insert under the U6 promoter (Sigma-Aldrich, St. Louis, MO, USA). Two shRNAs targeting exon 3 of *FLG2* gene (shFLG2) were tested (shFLG2a sequence: 5′-GATGATATTCAAGCTGACTAT-3′, nucleotide position 301–321 on the mRNA sequence (NCBI reference NM_001014342.2); shFLG2b sequence: 5′-CAGTTGGGAAAGAAGGAAAGATTT-3′, nucleotide position 763–783). A nontarget shRNA that does not target any known human gene (shc) was used as a control (5′-CCGGCAACAAGATGAAGAGCACCAACTC-3′).

### Keratinocyte culture and transduction

Primary normal human keratinocytes were obtained from abdominal dermolipectomy of three different healthy subjects who had given their informed consent. They were cultured and transduced with the lentivirus containing either shFLG2a, shFLG2b or shc at a multiplicity of infection of 10 in the presence of 4 *μ*g/ml of protamine sulfate, as described previously.^6^ After selection in the presence of puromycin, keratinocytes were used to produce RHE on polycarbonate filters as described previously.^25^ Briefly, cells were harvested by trypsinization, and 350 000 cells in ice-cold EpiLife medium (Invitrogen Life Technologies) containing 1.5 mM calcium were seeded on polycarbonate culture inserts (area of 0.63 cm^2^ with pores 0.4 *μ*m in diameter; Merck Millipore, Bedford, MA, USA). After 24 h of incubation at 37°C in a humidified atmosphere containing 5% CO_2_, cells were exposed to the air–liquid interface, and 50 *μ*g/ml vitamin C (Sigma-Aldrich) and 10 ng/ml keratinocyte growth factor (Sigma-Aldrich) were added to the medium in the lower compartment. The medium was renewed every 2 days during the 10 days of air–liquid interface culture.

### Immunostaining

At day 10, RHEs were fixed with 4% formaldehyde-containing buffer, and paraffin embedded. After deparaffinization and hydration, sections were blocked with PBS containing 2% bovine serum albumin and incubated with primary antibodies (Supplementary Table S1). After incubation with the corresponding secondary antibody (Alexa Fluor 555 anti-Rabbit IgG, Alexa Fluor 555 or 488 anti-Mouse IgG, Alexa Fluor 555 or 488 anti-Goat IgG, Invitrogen Life Technologies), nuclei were stained with DAPI (Sigma-Aldrich) and the slides were observed with a Nikon Eclipse 80i fluorescence microscope equipped with a Nikon DXM 1200 digital camera (Nikon, Tokyo, Japan).

### Morphological analysis

Epidermal thickness was assessed using images of hematoxylin–eosin-stained sections of paraffin-embedded RHEs, counting three fields per slide. Experiments were performed with keratinocytes from the three volunteers. Transmission electron microscopy analysis was carried out as previously described.^6^ Briefly, samples were fixed with 2.5% glutaraldehyde-formaldehyde solution, washed with Sorensen phosphate buffer, post-fixed with 1% OsO_4_, dehydrated and embedded in EmBed 812 resin (Electron Microscopy Sciences, Hatfield, PA, USA). Ultrathin sections were mounted on 100-mesh collodion-coated copper grids and post-stained with 3% uranyl acetate and 8.5% lead citrate before being examined with a Hitachi HT7700 electron microscope (Hitachi, Tokyo, Japan).

### Western blotting

At day 10, RHEs were lysed in 100 *μ*l of Laemmli buffer. Total epidermal proteins were separated on acrylamide gels and immunodetected with the primary antibodies and peroxidase-conjugated secondary antibodies (Goat anti-rabbit IgG-HRP and swine anti-goat IgG-HRP, SouthernBiotech, Birmingham, AL, USA; goat anti-mouse IgG-HRP, Bethyl Laboratories, Montgomery, TX, USA). Reaction products were detected by chemiluminescence with the ECL kit (Pierce/Thermo Scientific, Rockford, IL, USA). Quantity One 1-D Analysis Software (Bio-Rad Laboratories, Hercules, CA, USA) was used to quantify immunoreactive bands on western blot films after scanning. Signals were normalized to glyceraldehyde 3-phosphate dehydrogenase immunodetection.

### Reverse transcription-quantitative PCR

Reverse transcription (RT) was performed as previously described.^6^ The primers used are listed in the Supplementary Table S2. Only Ct values between 17 and 26 were considered. Relative levels of gene expression among samples were determined with the ΔΔ cycle threshold method. TATA box binding protein (TBP) gene expression was used for normalization.

### Analysis of Lucifer yellow permeability

At day 10, 200 *μ*l of 1 mM Lucifer yellow (Sigma-Aldrich) was added onto the SC of the RHEs. After incubation at 37°C for 6 h, the RHEs were fixed in 4% formaldehyde and embedded in paraffin. Sections were inspected under the fluorescence microscope. In parallel, the dye concentration in the culture medium was measured fluorometrically in a fluoroscan (Varioskan Flash, Thermo Fisher Scientific, Rockford, IL, USA) with excitation at 425 nm and emission at 550 nm.

### Determination of the pH at the RHE surface

At day 10, the pH at the RHE surface was determined with a pH electrode (Skin-pH-Meter PH 905, Courage+Khazaka Electronic GmbH, Köln, Germany).

### Determination of the urocanic acid and pyrrolidone carboxylic acid contents

At day 10, RHE specimens were lysed in 0.1 M KOH and amino acids were analyzed by HPLC as previously described.^8, 39^

### Statistical analysis

Statistical differences were determined with Student's *t*-test. A value of *P*<0.05 was considered statistically significant.

## Figures and Tables

**Table 1:** Comparison of filaggrin-2 and filaggrin knockdown in reconstructed human epidermis with AD skin

**Filaggrin-2 knockdown**	**Filaggrin knockdown**^a^	**AD**^b^
Normal stratum granulosum	Hypogranulosis	Hypogranulosis
Parakeratosis and a compact stratum corneum	Thin stratum corneum	Some parakeratosis
Vesicles in corneocytes	No vesicle in corneocytes	Vesicles in corneocytes
Normal corneocyte matrix	Disturbed corneocyte matrix	Disturbed corneocyte matrix
Normal outside-in SC permeability	Increased outside-in SC permeability	Increased outside-in SC permeability
Reduced NMF level	Reduced NMF level	Reduced NMF level
Increased pH	Unaffected pH	Increased pH
Decreased amounts of bleomycin hydrolase	Decreased amounts of bleomycin hydrolase	Decreased amounts of bleomycin hydrolase
Reduced proteolysis of filaggrin and hornerin precursors	Reduced expression of filaggrin-2 and hornerin	Reduced expression of filaggrin and hornerin abnormal (pro)filaggrin proteolysis
Reduced degradation of corneodesmosin	Increased expression of corneodesmosin	Persistence of corneodesmosomes
Reduced expression of loricrin	Reduced expression of loricrin	Reduced expression of loricrin
Abnormal expression of tight junction proteins	Abnormal expression of tight junction proteins	Abnormal expression of tight junction proteins

Abbreviation: NMF, natural moisturizing factor

See ^a^Pendaries *et al* ^6^ and ^b^Oyoshi *et al*^2^and Guttman-Yassky *et al*^40^ and references in the text

## Abbreviations

AD: atopic dermatitis
K: keratin
RHE: reconstructed human epidermis
RT-qPCR: reverse transcription-quantitative PCR
SC: *stratum corneum*
