# Supplementary material for: In a three-dimensional reconstructed human epidermis filaggrin-2 is essential for proper cornification
Source: Cell Death Dis. 2015 Feb 19;6(2):e1656–. doi: 10.1038/cddis.2015.29 (PMC4669814; doi:10.1038/cddis.2015.29)
Supplement: Supplementary Material [file cddis201529x1.doc]

**SUPPLEMENTARY TABLES**

**Table S**1: primary antibodies.

| **Antigen** | **Clone n° / name** | **Company** | **Dilution** |
| --- | --- | --- | --- |
| Filaggrin | AHF3 | Home made^1^ | 0.02 µg/ml |
| Filaggrin 2 | Polyclonal αhIFPS2 | Home made^2^ | 1:1000 |
| Hornerin | Polyclonal HPA031469 | Sigma-Aldrich | 1:500 |
| GAPDH | 6C5 | Santa Cruz Biotechnology | 1:500 |
| Involucrin | SY5 | Sigma-Aldrich | 1:1000 |
| Loricrin | AF62 | Covance | 1:10000 |
| Desmocollin 1 | Dsc1-U100 | Research Diagnostic Inc. | 1:50 |
| Desmoglein 1/2 | DG 3.10 | Progen | 1:500 |
| Claudin 1 | 1C5-D9 | Sigma-Aldrich | 1:500 |
| Keratin 10 | LH2 | Santa Cruz Biotechnology | 1:32000 |
| Keratin 14 | C8791 | Sigma-Aldrich | 1:500 |
| Bleomycin hydrolase | Polyclonal HPA039548 | Sigma-Aldrich | 1:1000 |
| Caspase 14 | D-10 | Santa Cruz Biotechnology | 1:100 |
| Calpain 1 | Polyclonal HPA005992 | Sigma-Aldrich | 1:250 |
| Corneodesmosin | F28-27 | Home made^3^ | 0.5 µg/ml |
| E-cadherin | Clone 36 | BD Transduction laboratories | 1:2500 |
| Kallikrein 7 | Ab28309 | Abcam | 1:2500 |
| Ki67 | M724029 | Dako | 1:500 |
| Active caspase 3 | AF835 | R&D systems | 1:500 |

^1^Simon M, Sebbag M, Haftek M, et al. Monoclonal antibodies to human epidermal filaggrin, some not recognizing profilaggrin. *J Invest Dermatol*, 1995; **105**:432-437. ^2^Wu Z, Hansmann B, Meyer-Hoffert U, Glaser R, Schroder J-M. Molecular identification and expression analysis of filaggrin-2, a member of the S100 fused-type protein family. *PLoS One* 2009; **4:**e5227. ^3^Guerrin M, Simon M, Montézin M, Haftek M, Vincent C, Serre, G. Expression cloning of human corneodesmosin proves its identity with the product of the S gene and allows improved characterization of its processing during keratinocyte differentiation. *J Biol Chem* 1998; **273**:22640-22647.

**Table S2: Sequences of qPCR primers.**

| **Gene** | **Forward** | **Reverse** |
| --- | --- | --- |
| ***FLG*** | 5’-GCAAGGTCAAGTCCAGGAGAA-3’ | 5’-CCCTCGGTTTCCACTGTCTC-3’ |
| ***FLG2*** | 5’-TCTGAAGAACCCAGATGATCCA-3’ | 5’-CATCAAAAGAAACTCAGTAAAGTCCAA-3’ |
| ***HRNR*** | 5’-AGGACAGGGCTATAGTCAGCA-3’ | 5’-CCGAAGCGTGATGGGAGG-3’ |
| ***IVL*** | 5’-GGGTGGTTATTTATGTTTGGGTGG-3’ | 5’-GCCAGGTCCAAGACATTCAAC-3’ |
| ***LOR*** | 5’-CGAAGGAGTTGGAGGTGTTT-3’ | 5’-ACTGGGGTTGGGAGGTAGTT-3’ |
| ***DSC1*** | 5’-CATGGGTGGTCAGCCTTTCGGT-3’ | 5’-TCCTGATCCTGTACCTTCATTGGCA-3’ |
| ***DSG1*** | 5’-GAAGGCAGAAACGTGAATGGA-3’ | 5’-TTTTGGCGATTGGGTTCCT-3’ |
| ***CLDN1*** | 5’-TGGCATGAAGTGTATGAAGTGCTT-3’ | 5’-CCCCAATGACAGCCATCCT-3’ |
| ***K10*** | 5’-TGATGTGAATGTGGAAATGAATGC-3’ | 5’-GTAGTCAGTTCCTTGCTCTTTTCA-3’ |
| ***K14*** | 5’-CTCATCCTCCCGCTTCTCCT-3’ | 5’-AAAGCCACTACCAAAGCTGCT-3’ |
| ***BLMH*** | 5’-GTGGTGGACAGGAAGCATGT-3’ | 5’-TCCTTTGCAGCTACGTCAGG-3’ |
| ***CASP14*** | 5’-TGCACGTTTATTCCACGGTA-3’ | 5’-TGCTTTGGATTTCAGGGTTC-3’ |
| ***CAPN1*** | 5’-CAAACACCCCTCCCCCAGGATGT-3’ | 5’-CGCACCCGCAGCTGCTCATA-3’ |
| ***CDSN*** | 5’-ACTGCTGCTGGCTGGTCT-3’ | 5’-AGAGCTTCTGGCACTGGAAA-3’ |
| ***CDH1*** | 5’-CTGCTGCTCTTGCTGTTTCTTC-3’ | 5’-CTCCGCCTCCTTCTTCATCATA-3’ |
| ***TBP*** | 5’-TCAAACCCAGAATTGTTCTCCTTAT-3’ | 5’-CCTGAATCCCTTTAGAATAGGGTAGA-3’ |
| ***KLK7*** | 5’-CCCCTGGAACCACCTGTACT-3’ | 5’-GTCCCCCTGAGTCACCATT-3’ |

**SUPPLEMENTARY LEGENDS**

**Figure S1. Expression of filaggrin-2 in skin and in RHE produced with keratinocytes from two individuals.** **(a)** Normal human skin and fully differentiated RHE were analyzed by indirect immunofluorescence with a monoclonal antibody directed to profilaggrin and filaggrin (pro(FLG)), and with a polyclonal antibody directed to filaggrin-2 (FLG2), as indicated. DNA was labeled with 4’,6’-diamidino-2-phenylindole (in blue). Scale bar, 50 µm. (**b and c**) Fully differentiated shc-RHE produced with keratinocytes from two different volunteers (RHE-2 and RHE-3) were analyzed by qRT-PCR (**b**) and by Western blotting performed with anti-FLG2 and anti-glyceraldehyde 3-phosphate dehydrogenase (GAPDH) antibodies (**c**).

**Figure S2. In RHE produced with shFLG2b-treated keratinocytes, expression of filaggrin-2 is greatly reduced and epidermal morphology is altered. (a)** At day 10, shc- and shFLG2b-RHE were analyzed by Western blotting with anti-filaggrin-2 (FLG2) and anti–glyceraldehyde 3-phosphate dehydrogenase (GAPDH) antibodies. **(b)** Sections of paraffin-embedded shc- and shFLG2b-RHE at day 10 were stained with hematoxylin and eosin. Bar = 50 µm.

**Figure S3. Filaggrin-2 knockdown modifies the keratinocyte proliferation rate. (a)** Equal number of shc- and shFLG2-treated keratinocytes (1x10^6^ cells) were seeded in 150 cm² flasks at day 0 (D0) and cultured in Complete DermaLife medium for three days. At day 3 (D3) cells were counted. (**b)** Staining of Ki67 (red) in keratinocytes of the basal layers of shc- and shFLG2-RHE at day 10. DNA was labeled with 4’,6’-diamidino-2-phenylindole (in blue). The thin line indicates the epidermis/polycarbonate membrane junction. Bars = 30 µm. Please note the non-specific labeling of the polycarbonate membrane. (**c**) The number of Ki67 positive (Ki67+) and negative (Ki67-) cells was quantified and is indicated as well as the total number of basal cells observed. The differences between shc- and shFLG2-RHEs were statistically significant as shown by the Pearson’s Chi-squared test. The data are representative of two different experiments performed with keratinocytes from two different donors.
