# Supplementary figures and images for: In a three-dimensional reconstructed human epidermis filaggrin-2 is essential for proper cornification
Source: Cell Death Dis. 2015 Feb 19;6(2):e1656–. doi: 10.1038/cddis.2015.29 (PMC4669814; doi:10.1038/cddis.2015.29)

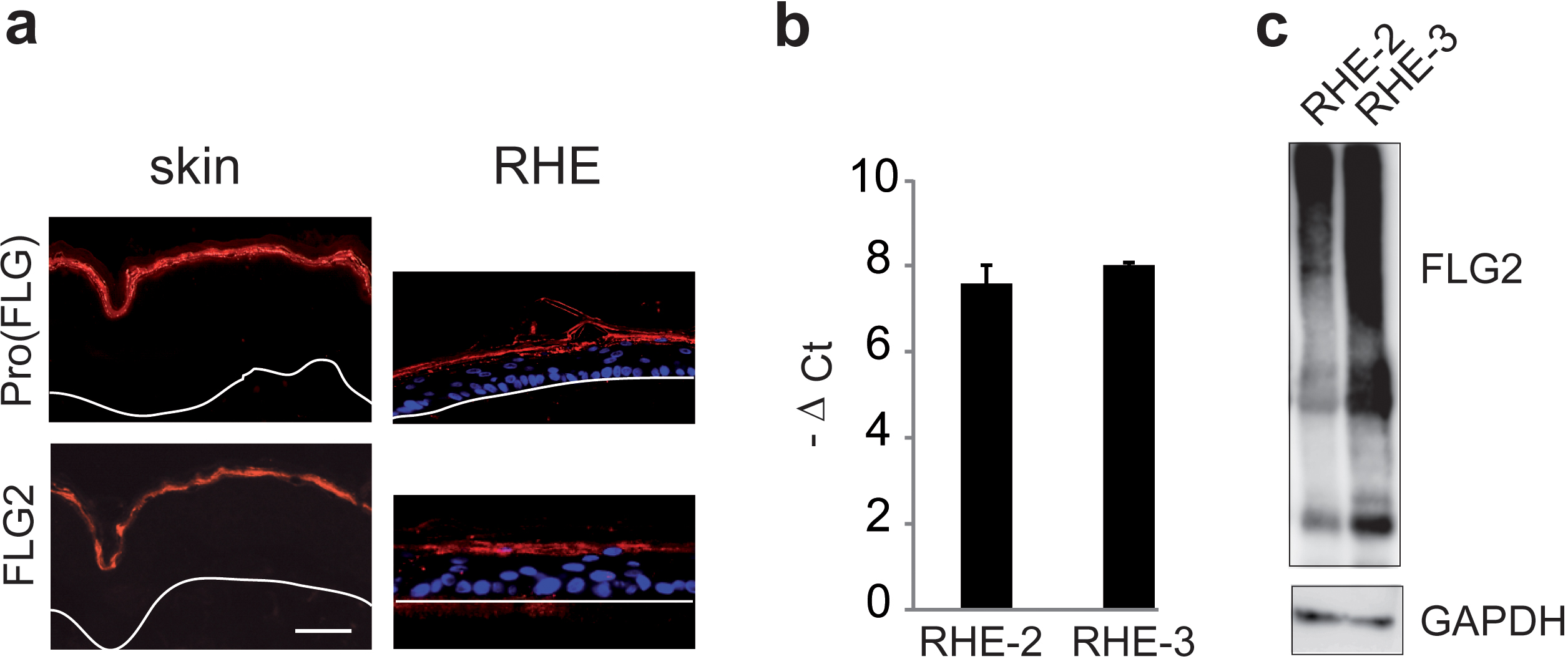

Supplement: Supplementary Figure S1 [file cddis201529x2.tif]

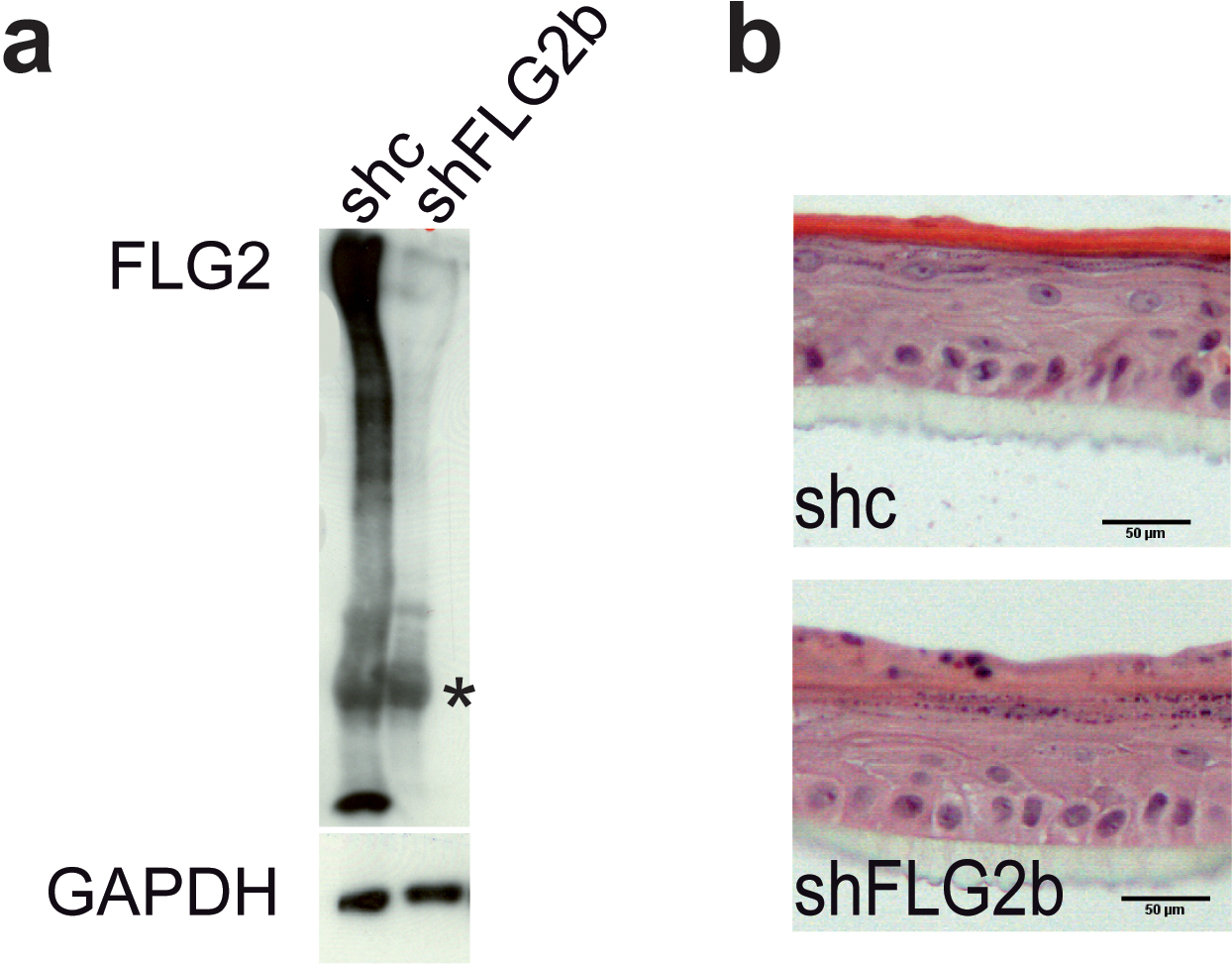

Supplement: Supplementary Figure S2 [file cddis201529x3.tif]

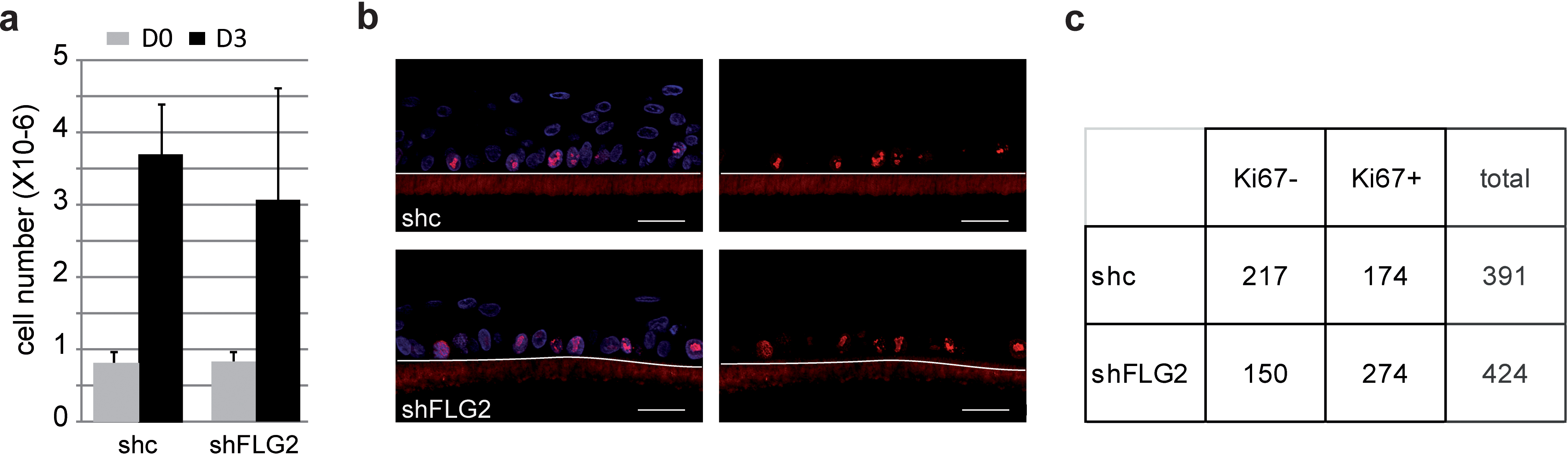

Supplement: Supplementary Figure S3 [file cddis201529x4.tif]
